# Antiangiogenic VEGF Isoform in Inflammatory Myopathies

**DOI:** 10.1155/2013/219313

**Published:** 2013-06-12

**Authors:** Nila Volpi, Alessandra Pecorelli, Paola Lorenzoni, Francesco Di Lazzaro, Giuseppe Belmonte, Margherita Aglianò, Luca Cantarini, Fabio Giannini, Giovanni Grasso, Giuseppe Valacchi

**Affiliations:** ^1^Department of Medicine, Surgery and Neuroscience, University of Siena, Via A. Moro 2, 53100 Siena, Italy; ^2^Department of Molecular and Developmental Medicine, University of Siena, Via A. Moro 2, 53100 Siena, Italy; ^3^Child Neuropsychiatry Unit, University Hospital AOUS, Viale M. Bracci 16, 53100 Siena, Italy; ^4^Department of Life Sciences and Biotechnologies, University of Ferrara, Via L. Borsari 46, 44121 Ferrara, Italy; ^5^Department of Food and Nutrition, Kyung Hee University, Seoul 130-701, Republic of Korea

## Abstract

*Objective*. To investigate expression of vascular endothelial growth factor (VEGF) antiangiogenic isoform A-_165b_ on human muscle in idiopathic inflammatory myopathies (IIM) and to compare distribution of angiogenic/antiangiogenic VEGFs, as isoforms shifts are described in other autoimmune disorders. *Subjects and Methods*. We analyzed VEGF-A_165b_ and VEGF-A by western blot and immunohistochemistry on skeletal muscle biopsies from 21 patients affected with IIM (polymyositis, dermatomyositis, and inclusion body myositis) and 6 control muscle samples. TGF-**β**, a prominent VEGF inductor, was analogously evaluated. Intergroup differences of western blot bands density were statistically examined. Endomysial vascularization, inflammatory score, and muscle regeneration, as pathological parameters of IIM, were quantitatively determined and their levels were confronted with VEGF expression. *Results*. VEGF-A_165b_ was significantly upregulated in IIM, as well as TGF-**β**. VEGF-A was diffusely expressed on unaffected myofibers, whereas regenerating/atrophic myofibres strongly reacted for both VEGF-A isoforms. Most inflammatory cells and endomysial vessels expressed both isoforms. VEGF-A_165b_ levels were in positive correlation to inflammatory score, endomysial vascularization, and TGF-**β**. *Conclusions*. Our findings indicate skeletal muscle expression of antiangiogenic VEGF-A_165b_ and preferential upregulation in IIM, suggesting that modulation of VEGF-A isoforms may occur in myositides.

## 1. Introduction

Idiopathic inflammatory myopathies (IIM) encompass three subsets: dermatomyositis (DM), polymyositis (PM), and sporadic inclusion body myositis (IBM) with distinct immunopathological patterns. In DM, complement deposition on endomysial capillaries, loss of microvessels, and mainly perivascular inflammation are observed, whereas PM and IBM show a T-cell invasion of muscle cells associated with degenerative features in IBM. Recently, necrotizing autoimmune myopathy (NAM), with scarce or no inflammation, has been recognized as a fourth IIM entity [1]. The expression of inflammatory effectors in IIM is extensively investigated, since pharmacological targeting of immunoregulatory factors is increasingly recognized as an effective treatment tool [2–4].

Vascular endothelial growth factor (VEGF) family comprises a group of potent endothelial cell mitogens. VEGF-A, an angiogenic growth factor, proinflammatory mediator, and promoter of vascular permeability, is produced by various cell types, among which are myocytes and inflammatory cells. Its expression is transcriptionally regulated by multiple molecules: growth factors, hormones, and oncogenes [5]. Alternative splicing from eight exons within the VEGF-A gene generates a family of proteins, named according to their amino acid number, VEGF_xxx_. VEGF family encompasses proangiogenic_xxx_ and antiangiogenic_xxxb_ isoforms, originating by alternate splice site selection in the terminal exon. Molecules have identical length but differing C-terminal amino acid sequences and opposing biological effect on angiogenesis: proangiogenic VEGF_xxx_ isoforms originate by proximal splice site selection and antiangiogenic VEGF_xxxb_ isoforms by distal splice site selection [6]. VEGF-A_165b_ is the first identified antiangiogenic VEGF molecule [6]. Inhibition of angiogenesis by VEGF_xxxb_ isoforms is due to competitive receptor binding, inhibition of receptor phosphorylation, and downstream intracellular signalling [7].

In the present work, we investigated tissue localization of VEGF-A_165b_ and VEGF-A in IIM as well as transforming growth factor-*β* (TGF-*β*), involved in the angiogenic and proliferative processes and a prominent VEGF-A_165b_ inductor, along with pathological parameters of myositides.

## 2. Subjects and Methods

### 2.1. Patients and Diagnostic Procedures

Muscle samples were archival diagnostic biopsy specimens from twenty-one patients affected with IIM, diagnosed by current clinicopathological criteria [1], as DM (*n* = 8), PM (*n* = 6), or IBM (*n* = 7). Demographic data are presented in Table 1. As control samples, we processed biopsy muscle specimens (*n* = 6) from subjects complaining of muscle pain, or cramps, with normal or mildly increased levels of serum creatine kinase and normal or mild myopathic electromyographic findings. We selected as controls only subjects in which muscle histology, histochemistry, immunohistology, and ultrastructural morphology resulted as normal, ruling out inflammation, degeneration-regeneration, denervation, or changes suggestive of metabolic storage. All subjects signed an informed consent with allowance to scientific utilization of muscle samples for research purposes. Specimens were frozen in liquid nitrogen-cooled isopentane and stored at −80°C until use. Cryostat sections, 10 *μ*m thick, were submitted to diagnostic routine histological and histochemical stains. 

### 2.2. Western Blot Analysis

WB analysis in muscle samples from control subjects and IIM patients was performed following standard procedures. Muscle samples were lysed in radioimmunoprecipitation assay (RIPA) buffer; proteins (40 *μ*g load) were separated by SDS-PAGE 4–20% Tris-Glycine Pre-Cast Gel (Invitrogen S.R.L., Milan, Italy) and transferred to a nitrocellulose membrane. After blocking, samples were incubated with the primary antibodies for VEGF-A (cod. MAB3734; Millipore Corporation, Billerica, MA, USA), VEGF-A_165b_ (Abcam Plc, Cambridge, UK), TGF-*β* (Santa Cruz Biotechnology, Inc., Santa Cruz, CA, USA), and *β*-actin (Cell Signaling Technology, Inc., Danvers, MA, USA) and then with the appropriate secondary antibodies. Bound antibodies were visualized by enhanced chemiluminescence (ECL Detection Kit, GE Healthcare, Milan, Italy), and the band densities were quantified using NIH image software by using *β*-actin band for normalization.

### 2.3. Immunohistochemistry

Immunohistology for diagnostic analysis of inflammation (HLA-ABC, C5-b9, CD4, CD8, CD11b, CD20, CD45Ro—Dako, Glostrup, Denmark; Carpinteria, CA, USA) was carried out on 7 *μ*m thick cryostat sections on silane-coated slides (StarFrost; Knittel Gläser, Braunschweig, Germany). Immunolocalization of VEGF-A and VEGF-A_165b_ was performed on consecutive sections. Immunohistochemistry for TGF-*β* and endothelial marker CD31 (Dako), for vessel detection, were also carried out. Regenerating fibres were identified by antifoetal myosin heavy chain antibody (Novocastra Laboratories Ltd, Newcastle upon Tyne, UK). Analysis was performed by immunoperoxidase technique, by HRP-labeled polymer (Dako), and 3,3′-diaminobenzidine (Sigma-Aldrich, Milan, Italy) for visualization. Negative controls by omission of the primary antibody were performed.

### 2.4. Quantitative Analysis on Immunohistochemical Slides

Morphometry was carried out by Zeiss AxioPlan2 microscope equipped with AxioVision 4.6 software (Carl Zeiss Vision GmbH, Hallbergmoos, Germany). The endomysial vessel density, the degree of inflammation, and the entity of muscle regeneration were evaluated, as histopathological parameters of myositis.

Density of endomysial vessels was expressed as number/mm^2^ of muscle area by counting the whole sections immunostained for CD31. Inflammatory score was assessed on sections stained for marker of activated leukocytes CD45Ro. For each sample, three randomly selected fields at 100x magnification were analyzed by automatized colorimetric pixel evaluation, detecting the peroxidase reaction product. CD45Ro peroxidase labeled area and the area of the whole field were measured: inflammatory score was expressed as the CD45Ro+ percentage of the total area.

Regeneration index was expressed as the percentage of foetal myosin heavy chain reactive fibres by examination of at least 700 fibres.

### 2.5. Statistics

Data were evaluated by SPSS statistics software. IIM were examined as a whole versus controls and as single subgroups. Intergroup variabilities were analyzed by Mann-Whitney test for paired analysis, and analysis of variance was performed by Kruskal-Wallis test for multiple groups. Spearman rank test was used to analyze correlations between WB band densities and the other investigated pathological parameters. Significance was set at *P* < 0.05. Data were expressed as means ± SD.

## 3. Results

### 3.1. Western Blot

#### 3.1.1. VEGF-A and VEGF-A_165b_

**Figure 1:**
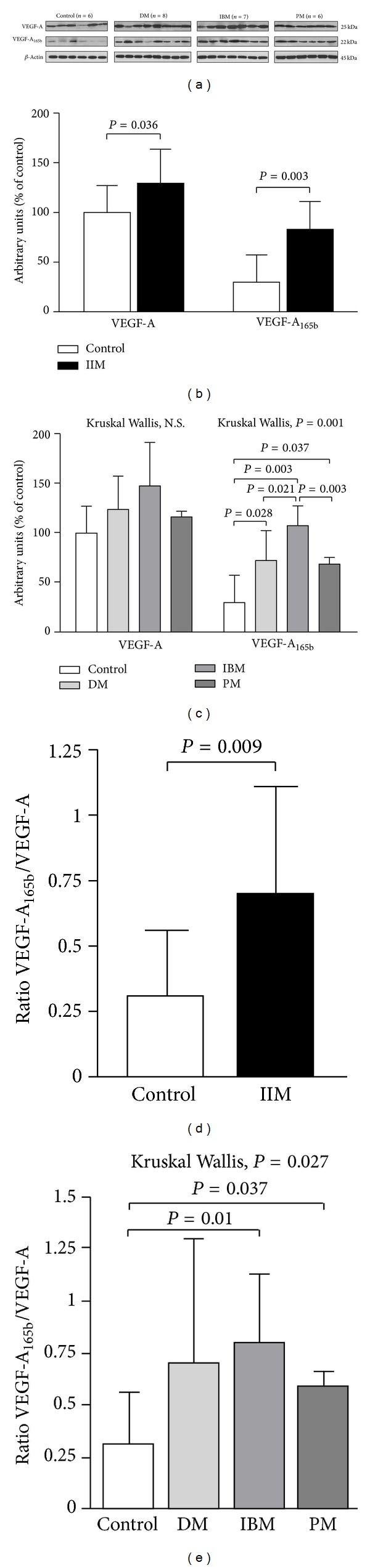
Increase of VEGF-A and VEGF-A_165b_ protein expressions in inflammatory myopathies. Representative VEGF-A and VEGF-A_165b_ western blots (a). Densitometric quantification of the VEGF-A and VEGF-A_165b_ bands is shown as ratio with the loading control *β*-actin in IIM (b) and in DM, IBM, and PM subsets (c). VEGF-A_165b_/VEGF-A ratio was obtained for all samples ((d), (e)). Data are expressed as arbitrary units from five independent experiments. Histograms represent mean values ± SD. All the statistical significant differences were reported.

The protein levels of both VEGF isoforms were higher in IIM subjects (Figure 1). An increase of circa 30% for VEGF-A and up to 2.5-fold for VEGF-A_165b_ versus controls (Figure 1(b)) was observed. VEGF-A_165b_ was significantly upregulated in the IIM subgroups DM, IBM, and PM versus controls; analysis of intergroup variance evidentiated significantly higher levels of VEGF-A_165b_ in IBM versus PM and DM (Figure 1(c)). The ratio VEFG-A_165b_/VEGF-A was significantly increased in IIM (Figure 1(d)), indicating a preferential upregulation of the antiangiogenic subunit: IIM 0.70 ± 0.40 (min. 0.15, max 2.09); ctrls: 0.31 ± 0.25 (min 0.03, max 0.68). PM and IBM subgroups were significantly different from controls (Figure 1(e)). 

#### 3.1.2. TGF-*β*

**Figure 2:**
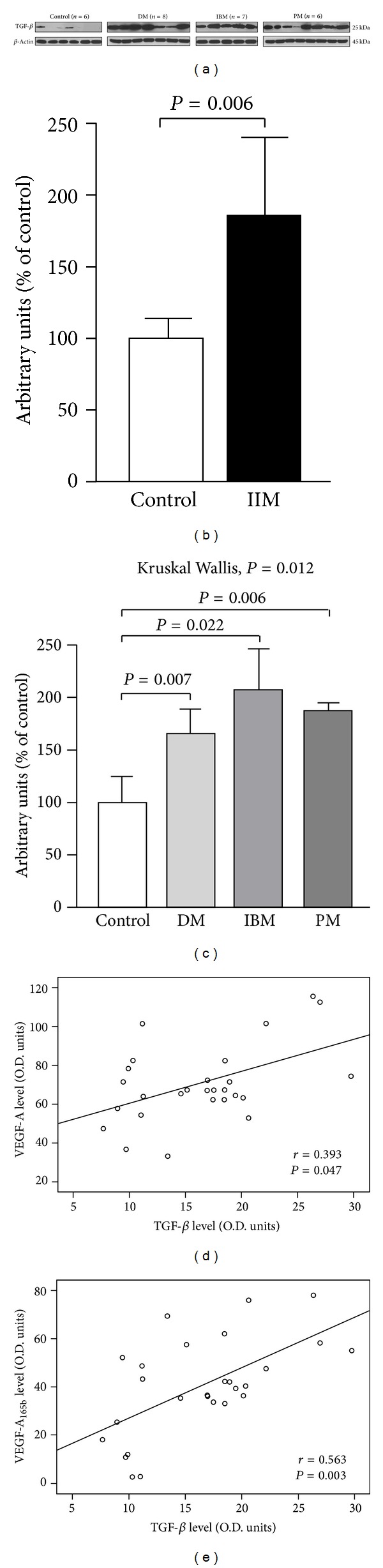
Increase of TGF-*β* protein expression in inflammatory myopathies. Representative TGF-*β* Western blots (a). Densitometric quantification of the TGF-*β* bands was obtained by *β*-actin normalization ((b), (c)). Histograms represent mean values ± SD from five independent experiments. All the statistical significant differences were reported. TGF-*β* levels were in positive correlation with VEGF-A (d) and VEGF-A_165b_ (e).

IIM samples displayed an almost 2-fold increase in TGF-*β* levels versus controls (Figure 2(b)), with statistical significance in all the IIM subsets (Figure 2(c)). TGF-*β* protein levels in IIM were positively related with both VEGF isoforms content (Figures 2(d) and 2(e)). 

### 3.2. Immunohistology

#### 3.2.1. VEGF-A, VEGF-A_165b_, and TGF-*β* Localization

**Figure 3:**
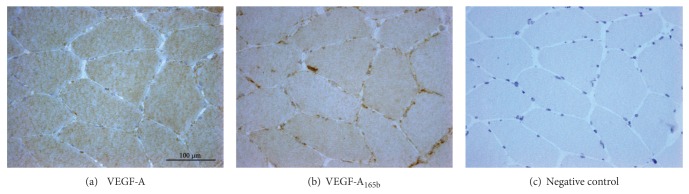
Immunohistological distribution of VEGF isoforms in control muscle. (a)–(c) Consecutive sections. Diffuse VEGF-A cytoplasmic stain of myofibers is observed (a); very mild cytoplasmic VEGF-A_165b_ reactivity, with stronger stain of endomysial sparse mononuclear cells and endomysial small vessels (b). No stain on negative control slide by omission of primary antibody (c).

**Figure 4:**
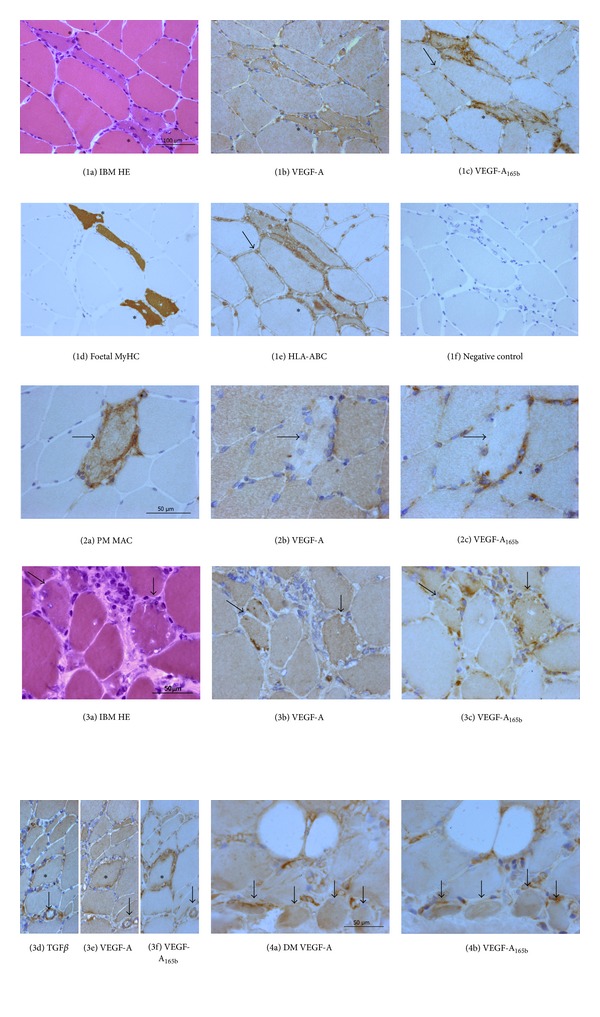
Representative images of immunohistological distribution of VEGF-A, VEGF-A_165b_, and TGF-*β* in inflammatory myopathies. (1a)–(1f) Consecutive sections, IBM. Regenerating fibres (asterisk), basophilic (1a) and reactive for foetal myosin heavy chain (1d), show VEGF-A (1b) upregulation, against basal expression of adjacent fibres. These fibres are strongly VEGF-A_165b_ reactive (1c), in contrast with low or null reactivity of adjacent fibres, and show highly reactive foci. Small endomysial vessels are diffusely VEGF-A and VEGF-A_165b_ reactive. A mild VEGF-A_165b_ upregulation (1c: arrow) is observed in nonregenerating fibres displaying sarcolemmal and cytoplasmic HLA-ABC neoexpression (1e: arrow). (1f) Negative control slide. (2a)–(2c) Consecutive sections, PM. Necrotic fibres, identified by deposits of the terminal complex of complement, or membranolytic attack complex (MAC) (2a: arrow), do not express neither VEGF-A (2b: arrow) nor VEGF-A_165b_ (2c: arrow), appearing as unreactive pale elements. Mononuclear cells surrounding and partially invading the necrotic fibre (2c: asterisk) are strongly reactive for VEGF-A_165b_. (3a)–(3c) Consecutive sections, IBM. Fibres with rimmed vacuoles (3a, arrows) show VEGF-A upregulation (3b, arrows), with occasional highly reactive foci, and VEGF-A_165b_ upregulation with multiple spots of intense reactivity (3c, arrows). Mononuclear infiltrates show a scarce reactivity for VEGF-A (3b), whereas they strongly express VEGF-A_165b_. (3d)–(3f) IBM. TGF-*β* (3d) is expressed on a large endomysial vessel (arrow) and inflammatory cells. Muscle fibres of smaller diameter show higher reactivity. On nonadjacent serial sections, localization of both VEGF isoforms is observed ((3e)-(3f)) in the vessel. The asterisk identifies a fibre surrounded by inflammatory cells. (4a)-(4b) Consecutive sections, DM. Perifascicular atrophic fibres (arrows) show increased reactivity for angiogenic (3a) and antiangiogenic (3b) VEGF isoforms. Mononuclear cells also react for both isoforms.

Muscle fibres showed a constitutive diffuse cytoplasmic expression of VEGF-A, either in controls (Figure 3(a)) and in IIM (Figures 4(1b); 4(2b); 4(3b); 4(4a)). Cytoplasmic localization of VEGF-A_165b_ was very faint in control muscle, where endomysial vessels appear the main source of the protein (Figure 3(b)) and in morphologically normal fibres of IIM (Figures 4(1c); 4(2c); 4(3c); 4(4b)). In myositis, atrophic and regenerating fibres (Figure 4(1a)), identified by foetal myosin (Figure 4(1d)), were strongly reactive for either isoforms. A milder VEGF-A_165b_ upregulation was observed in most nonregenerating, morphologically normal fibres (Figure 4(1c)), with neolocalization of MHC I molecule HLA-ABC (Figure 4(1e)). Necrotic fibres, identified by deposits of the terminal complex of complement (Figure 4(2a)), displayed no VEGF or VEGF-A_165b_ expression (Figures 4(2b)-4(2c)). Vacuolated fibres of IBM (Figures 4(3a)–4(3c)) strongly expressed VEGF-A_165b_ in adjacency of rimmed vacuoles, a pathological hallmark of IBM. Perifascicular atrophic fibres of DM also showed an increased expression of both VEGF isoforms (Figures 4(4a)-4(4b)). Areas of VEGF upregulation displayed a substantial VEGF-A/VEGF-A_165b_ co-localization but a stronger diffuse immunostain and a high occurrence of focal reactive deposits resulted distinctive VEGF-A_165b_ features (Figures 4(1b)-4(1c), 4(3b)-4(3c)). Both VEGFs were also expressed by endothelium of most capillaries (Figures 4(1b)-4(1c)) or larger endomysial vessels (Figures 4(3e)-4(3f)). Occurrence of mononuclear infiltrates with scarce VEGF-A stain was observed in subjects submitted to steroid treatment (Figure 4(3b)), whereas VEGF-A_165b_ expression was maintained (Figure 4(3c)). In biopsies performed prior to therapy, inflammatory cells strongly reacted for both VEGF isoforms (Figures 4(4a)-4(4b)). TGF-*β* was detected in inflammatory cells and muscle fibres in adjacency of infiltrates, as well as in vessel walls (Figure 4(3d)), in substantial colocalization with VEGFs (Figures 4(3e)-4(3f)). 

### 3.3. Quantitative Analysis

#### 3.3.1. Density of Endomysial Vessels

**Figure 5:**
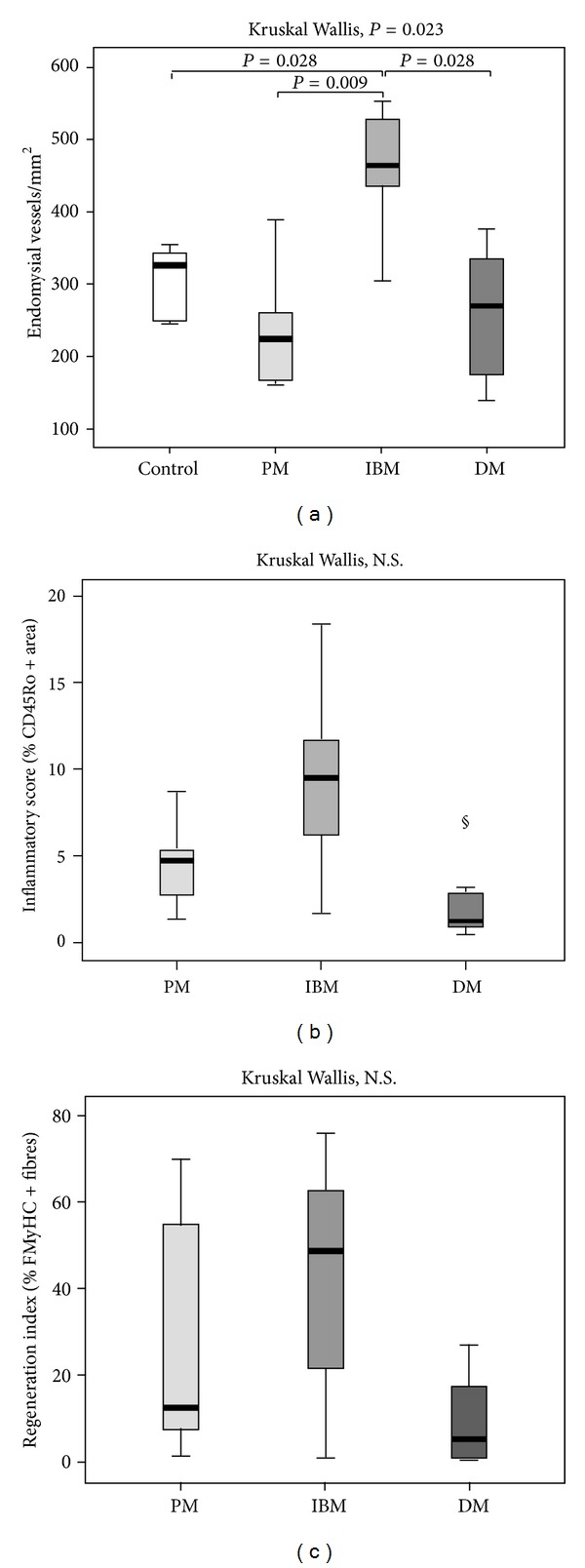
Quantitative analysis of pathological parameters in inflammatory myopathies. (a) A significant increase of the vessels density in IBM cases is detected. PM and DM cases showed a mild decrease of vessel density. (b) IBM cases showed the highest inflammatory score. § The DM outlier, with an unusually high inflammatory score, is a case of severe paraneoplastic DM, with high degree of myofiber necrosis and inflammation. (c) Regeneration index: the occurrence of regeneration showed a high variability among subjects, within the IIM subsets. The values are reported as means ± SD. All the statistical significant differences were reported.

Variations of endomysial vascularization were detected, accordingly to literature data. IBM cases showed a significantly higher vessel density than controls (Figure 5(a)). Analysis of variance among IIM subsets evidentiated a significantly higher endomysial vascularization in IBM versus PM and DM.

Endomysial vessel density in IIM was in positive correlation with VEGF-A (*r* = 0.585,  *P* = 0.017,) and VEGF-A_165b_ levels (*r* = 0.503,  *P* = 0.047), as well as with VEGF-A_165b_/VEGF-A ratio (*r* = 0.812,  *P* < 0.001).

#### 3.3.2. Inflammatory Score

IBM cases showed the highest degree of inflammation compared to the other groups (Figure 5(b)). Inflammatory score in IIM was in positive correlation with VEGF-A_165b_ levels (*r* = 0.630, *P* = 0.009), as well as with vessel density (*r* = 0.499, *P* = 0.049). 

#### 3.3.3. Regeneration Index

The occurrence of regenerating fibres was highly variable among and within the subgroups (Figure 5(c)): IBM and PM showed higher regeneration percentage as compared to DM. 

The regeneration index was positively related to TGF-*β* protein level in the whole group of IIM (*r* = 0.473,  *P* = 0.03) and to VEGF-A_165b_/VEGF-A ratio (*r* = 0.523,  *P* = 0.012).

## 4. Discussion

The present report demonstrates that VEGF-A_165b_ is expressed in human skeletal muscle and its level robustly increases in IIM. 

Constitutive expression of VEGF by myocytes is found to be essential for regulation of capillarity [8] and, in aged humans, the lower density of endomysial capillaries is associated with VEGF local decrease [9]. Therefore, the VEGF increase that we report in IIM is not biased, but rather stressed, by the younger median age of our noninflammatory controls.

Increase of VEGF *in toto* in skeletal muscle following injury, in association with fibres regeneration, is documented [10]. 

A recent fundamental study highlighted increased muscle expression of VEGF-A in IIM, in correlation with clinicopathological stages of disease, and it also specifically addressed the issue of therapy influence [11]. Glucocorticoid treatment (3–6 months) in PM and DM lowered VEGF-A expression, which stayed anyway significantly higher than in controls, by decreasing the number of VEGF-A reactive capillaries and mononuclear inflammatory cells, as we also observed in our treated patients. Therefore a role of steroids is likely also in the modulation of muscle VEGF-A_165b_; however, the highest levels of both VEGF isoforms were detected in our group of IBM patients, typically unresponsive to steroids [4], all submitted to 1–4 months glucocorticoid treatment, prior to biopsy and histological diagnosis. 

The constitutive expression of antiangiogenic VEGF isoforms is highly variable (reviewed by [12]). They are downregulated in highly angiogenic tissues, such as placenta [13], but they may reach 90% of the total VEGF protein in colon [14].

A previous study investigating antiangiogenic isoforms of VEGF-A in human muscle did not detect VEGF-A_165b_, basally or after submaximal exercise, in healthy subjects, by PCR [15], whereas previous PCR [6], and ELISA [16] identifications of VEGF_165b_ in skeletal muscle are reported. As skeletal muscle is richly vascularized, either Western blot or ELISA, at the protein level, and PCR, at the mRNA level, do not rule out detection of endothelial molecules. As we agree that, within normal muscle, the antiangiogenic VEGF subunit is mainly expressed by endothelial cells, the events of inflammation and coexisting atrophy/regeneration in IIM seem to trigger a substantial VEGF_165b_ upregulation in muscle. A basal constitutive VEGF-A_165b_ synthesis by myocytes, against a diffusion from adjacent inflammatory cells, is also suggested by the absent stain, for either VEGF isoforms, in necrotic fibres. The specificity of the utilized VEGF-A_165b_ antibody, directed against the antiangiogenic COOH-terminal sequence, was assessed by complete lack of recognition of angiogenic VEGF-A_165_ protein isoform by WB, as documented by manufacturer (http://www.abcam.com/VEGF-165B-antibody-MRVL56-1-ab14994.html). The differences in the staining pattern of the two isoforms further support antibody specificity.

Studies addressing the alternate distal/proximal site splicing in human disease describe a splicing switch to pro-angiogenic isoform in angiogenesis-associated disorders, such as solid tumors and diabetic retinopathy [17, 18]. Opposingly, a selective upregulation of VEGF-A_165b_ is reported in retinal detachment associated with proliferative vitreoretinopathy, glaucoma [19], and fibrosing autoimmune disorder systemic sclerosis [20, 21], where decrease of angiogenesis occurs.

In our IIM samples, a local VEGF-A_165b_ preferential upregulation is suggested by the increased VEGF-A_165b_/VEGF-A ratio.

Research data on VEGF-A_165b_ offer clues to elucidate its increase in IIM: growth factors and related signal pathways act on VEGF_xxx_/VEGF_xxxb_ alternative splicing [5, 17], and pleiotropic fibrogenic TGF-*β* is a key factor in switch to distal site splice selection for synthesis of VEGF-A_165b_ [5, 20]. In our IIM samples, as previously described [2, 22], TGF-*β* was upregulated, and we observed a positive correlation TGF-*β*/VEGF-A_165b_ by western blot. Therefore, the peculiar VEGF-A_165b_ staining of degenerating/regenerating fibres may be linked to molecular events of muscle regeneration, where upregulation of TGF-*β* occurs [23], as supported by correlation of extent of regeneration and VEGF-A_165b_/VEGF-A ratio. Nevertheless, the lack of a direct correlation VEGF-A/regeneration index and a milder VEGF-A_165b_ localization in nonregenerating fibres neoexpressing major histocompatibility complex-I, a change consistent with myofiber activation (reviewed in [24]), suggest that other factors contribute significantly to VEGF-A_165b_ upregulation in IIM. Immunohistology shows that TGF-*β* reactive endothelium and inflammatory cells are also a prominent source of VEGF-A_165b_. The finding is associated to inflammatory score/VEGF-A_165b_ and both VEGF isoforms/vessel density positive correlations.

Though our patients' subgroups are numerically too small to allow general conclusions, IBM higher inflammatory score and density of endomysial vessels, an acknowledged pathological feature of IBM [3], which resulted in our study strictly related to VEGF-A_165b_/VEGF-A ratio, appear to concur to higher VEGF-A_165b_ expression. This apparently controversial issue might depend on a negative feedback triggered by VEGF-A_165b_. In fact, as it has been proposed [6, 25], VEGF-A_165b_ can inhibit VEGF-A-mediated proliferation and migration of endothelial cells, via a counterregulatory mechanism, limiting the rate of *de novo *muscle capillarization.

Moreover, hypoxia, a strong VEGF modulating agent [14, 21, 26, 27], due to microvascular involvement, has a role in all the major IIM subsets by activating circuitries of cytokines, adhesion molecules, and leukocyte recruitment [3]. Skeletal muscle reacts with increased VEGF-A gene expression to hypoxia caused by acute exercise [27]. As an inductor of TGF-*β* [28], hypoxia might result in an additional causative factor of local VEGF-A_165b_ increase in IIM.

Interestingly, besides antiangiogenic effect, a cytoprotective activity of VEGF-A_165b_ against ischaemic damage has been recently documented on colon epithelium [14] and on retinal epithelium and endothelium [29], so that its compound biological effects still need to be elucidated. 

## 5. Conclusions

Local VEGF antiangiogenic switch in inflammation and necrosis/regeneration of skeletal muscle is likely to depend on a cohort of humoral and cellular effectors, possibly involved in tissue protection against inflammatory process. 

As therapeutic modulation of VEGF isoforms is currently investigated in cancer [12, 14] and in angiogenic eye disorders [17], and considering a potential development in autoimmune disorder systemic sclerosis [20, 21], further studies are needed for a complete understanding of the balance between antiangiogenic and proangiogenic VEGF isoforms in inflammatory myopathies. 

## Supplementary Material

Control case: VEGF_A_ expressed by muscle fibres IBM (adjacent section of Fig 3 1a-e) negative control by omission of primary antibodies. No stain is present on fibres, vessels, and connective tissue.

## Figures and Tables

**Table 1:** Demographic data.

	PM	DM	IBM	Controls
No. of subjects	6	8	7	6
Gender: M, F	1, 5	3, 5	6, 1	4, 3
Biopsy site	Vastus lateralis: 4 Deltoid: 2	Vastus lateralis: 6 Deltoid: 2	Vastus lateralis: 6 Brachial biceps: 1	Vastus lateralis: 7
Age at biopsy, years (range)	61.33 ± 7.87 (53–63)	58.25 ± 12.07 (42–72)	72.43 ± 7.91 (61–85)	45.42 ± 23.22 (18–73)
Symptoms before biopsy, months	12.5 ± 5.6	14.6 ± 9	40 ± 11.8	—
Immunotherapy before biopsy (1–4 months) prednisolone: 5–10 mg/day	3/6	4/8	7/7	—
