# Supplementary material for: Antiangiogenic VEGF Isoform in Inflammatory Myopathies
Source: Mediators Inflamm. 2013 Jun 12;2013:219313. doi: 10.1155/2013/219313 (PMC3694558; doi:10.1155/2013/219313)
Supplement: Supplementary file 1 — Control case: VEGFA expressed by muscle fibres IBM (adjacent section of Fig 3 1a-e) negative control by omission of primary antibodies. No stain is present on fibres, vessels, and connective tissue. [file 219313.f1.doc]

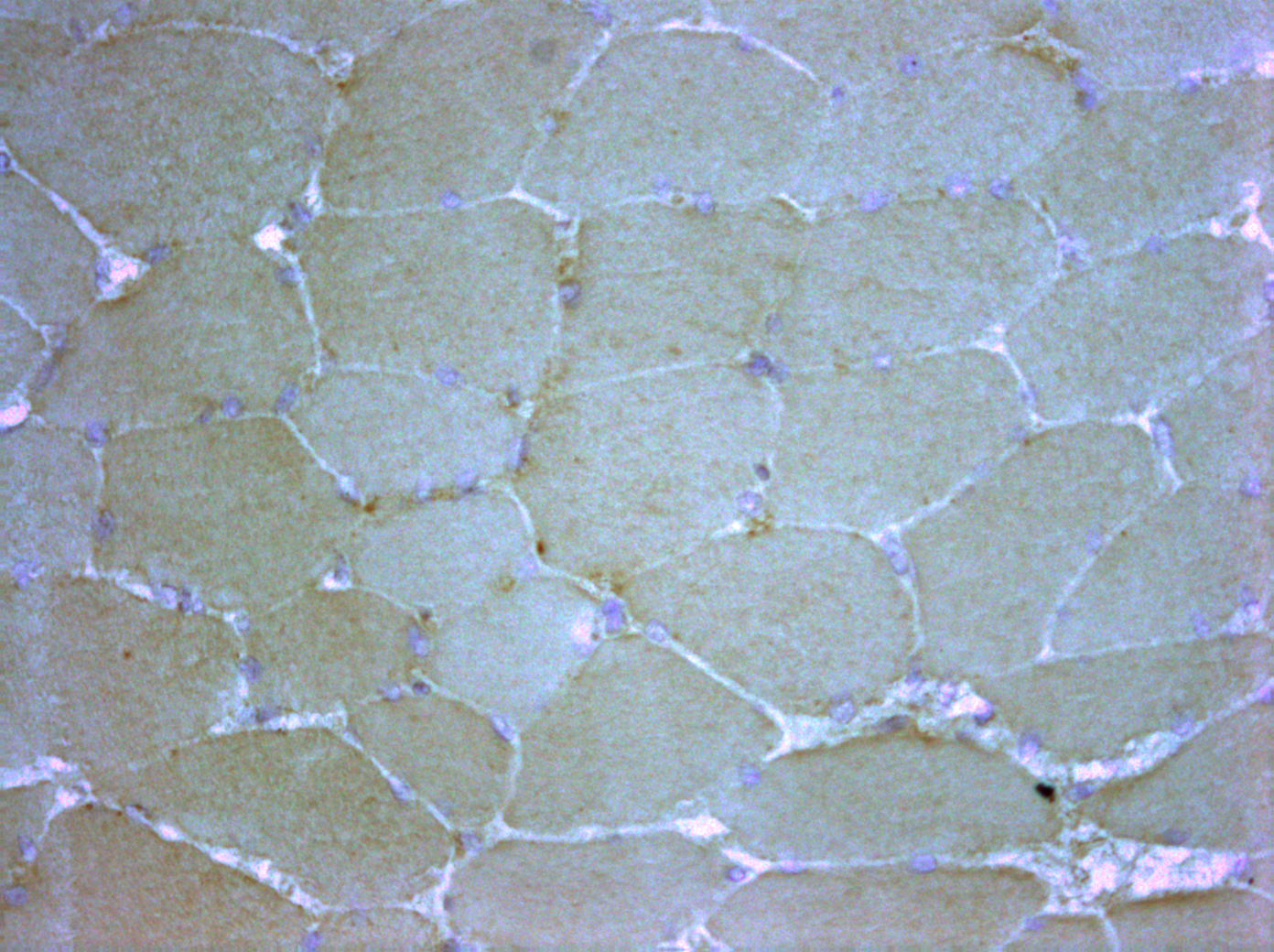


**Control case: VEGF_A_ expressed by muscle fibres**

**
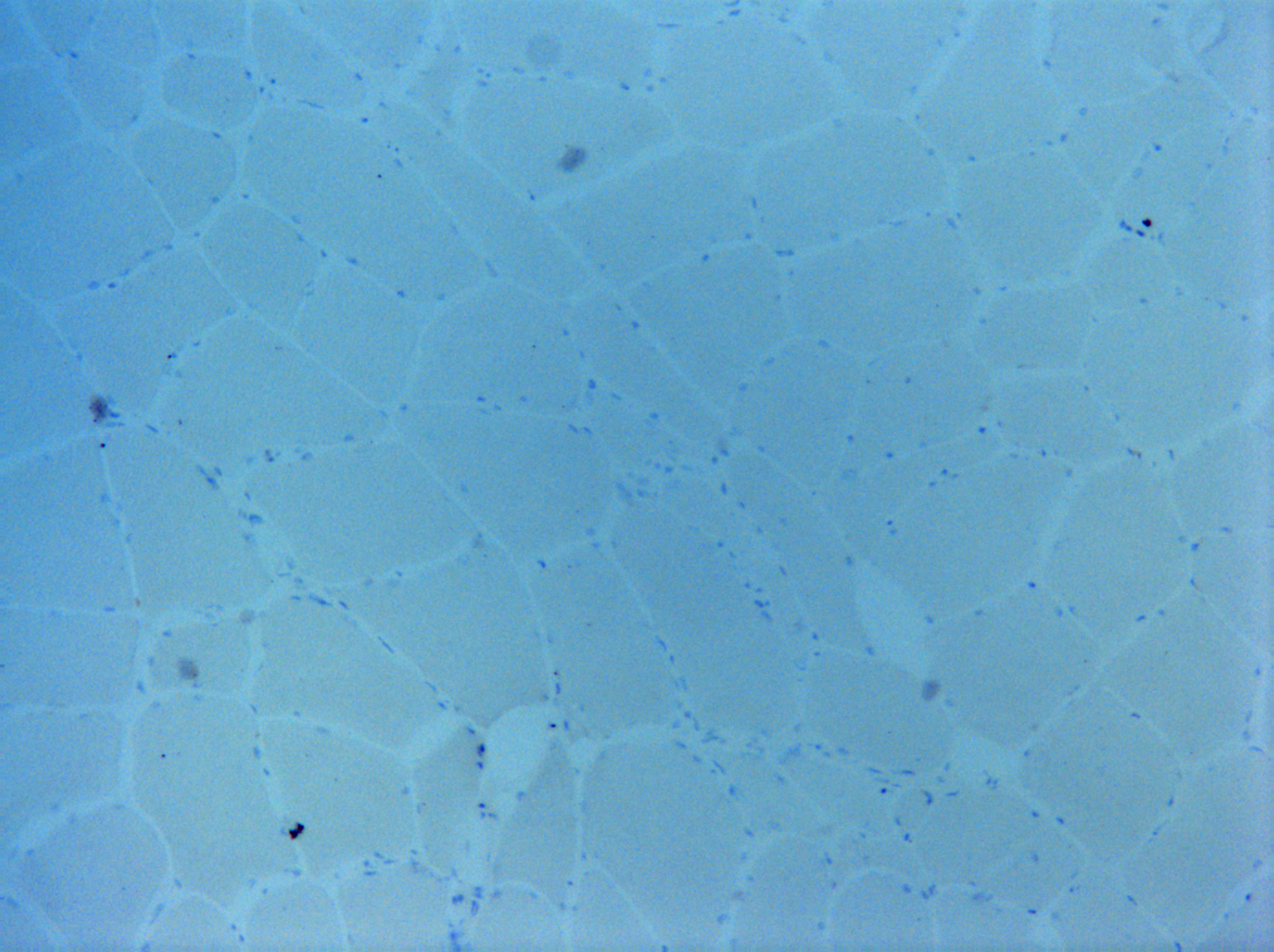
**

**IBM (adjacent section of Fig 3 1a-e) negative control by omission of primary antibodies.**

**No stain is present on fibres, vessels, and connective tissue.**
